# Cell density and extracellular matrix composition mitigate bacterial biofilm sensitivity to UV-C LED irradiation

**DOI:** 10.1007/s00253-024-13123-4

**Published:** 2024-04-05

**Authors:** Maritxu Labadie, Frédéric Marchal, Nofel Merbahi, Elisabeth Girbal-Neuhauser, Catherine Fontagné-Faucher, Claire-Emmanuelle Marcato-Romain

**Affiliations:** 1 Université de Toulouse, UPS, IUT Paul Sabatier, LBAE EA 4565 (Laboratoire de Biotechnologies Agroalimentaire Et Environnementale), 24 Rue d’Embaquès, Auch, F-32000 France; 2 Université de Toulouse, UPS, INPT, LAPLACE UMR 5223 (Laboratoire Plasma Et Conversion d’Energie), 118 Route de Narbonne, Toulouse, F-31062 France

**Keywords:** Biofilms, Extracellular polymeric substances, 280 nm UV-C LED, Disinfection, *Pseudomonas aeruginosa*, *Leuconostoc citreum*

## Abstract

**Abstract:**

Ultraviolet-C light-emitting diodes (UV-C LEDs) are an emerging technology for decontamination applications in different sectors. In this study, the inactivation of bacterial biofilms was investigated by applying an UV-C LED emitting at 280 nm and by measuring both the influence of the initial cell density (load) and presence of an extracellular matrix (biofilm). Two bacterial strains exposing diverging matrix structures and biochemical compositions were used: *Pseudomonas aeruginosa* and *Leuconostoc citreum*. UV-C LED irradiation was applied at three UV doses (171 to 684 mJ/cm^2^) on both surface-spread cells and on 24-h biofilms and under controlled cell loads, and bacterial survival was determined. All surface-spread bacteria, between 10^5^ and 10^9^ CFU/cm^2^, and biofilms at 10^8^ CFU/cm^2^ showed that bacterial response to irradiation was dose-dependent. The treatment efficacy decreased significantly for *L. citreum* surface-spread cells when the initial cell load was high, while no load effect was observed for *P. aeruginosa*. Inactivation was also reduced when bacteria were grown under a biofilm form, especially for *P. aeruginosa*: a protective effect could be attributed to abundant extracellular DNA and proteins in the matrix of *P. aeruginosa* biofilms, as revealed by Confocal Laser Scanning Microscopy observations. This study showed that initial cell load and exopolymeric substances are major factors influencing UV-C LED antibiofilm treatment efficacy.

**Key points:**

• *Bacterial cell load (CFU/cm*^*2*^*) could impact UV-C LED irradiation efficiency*

• *Characteristics of the biofilm matrix have a paramount importance on inactivation*

• *The dose to be applied can be predicted based on biofilm properties*

**Graphical Abstract:**

**Figure:**
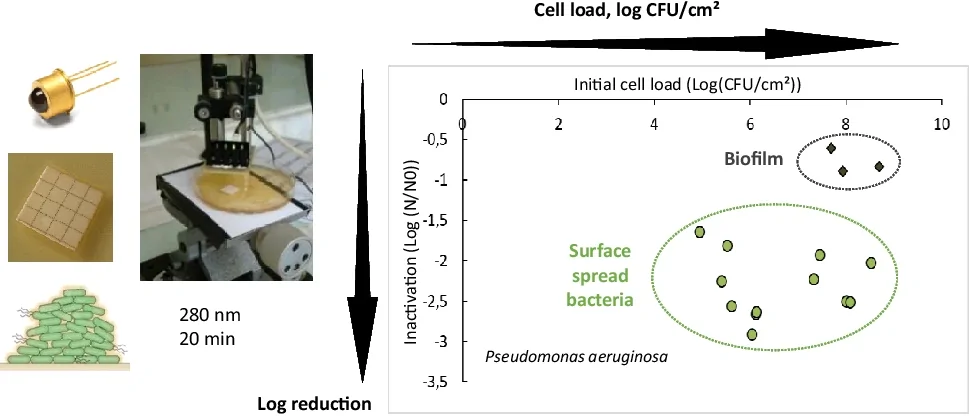

**Supplementary Information:**

The online version contains supplementary material available at 10.1007/s00253-024-13123-4.

## Introduction

Biofilms are described as microbial cells aggregated together through a matrix of extracellular polymeric substances (EPS) and attached to a surface. The composition of the matrix varies according to the inherent bacterial strains; nevertheless, the main components are often described as a complex mixture of polysaccharides, proteins, and extracellular DNA (Flemming 2011). This biofilm matrix ensures, among other functions, a well-known protective role towards bacterial cells, such as providing better resistance to disinfectant treatments, antibiotic applications, and other abiotic stresses compared to planktonic free-living bacterial cells (Flemming and Wingender 2010). The persistence of biofilms attached to surfaces can hence lead to considerable sanitary concerns, particularly in the food and water distribution sectors, such Listeria or Legionella’s induced diseases, respectively. Surface decontamination is obviously a priority in these sectors and has been dealt with for many years, notably by using efficient chemical biocides with the risk of spreading out potentially harmful by-products. In this context, the emergence of resistant bacterial biofilms able to withstand conventional antimicrobial treatments and the need for more sustainable decontamination technologies have both encouraged recent developments regarding eco-friendly and innovative strategies to control biofilms.

Ultraviolet C light irradiation is a decontamination technology widely used for water, air, and surface disinfection (Hijnen et al. 2006), and now also emerging for food preservation (Keklik and Demirci 2014). The germicidal wavelength range is approximately between 200 and 300 nm, and UV-light for decontamination purposes is generated from conventional low-pressure mercury lamps, which emit at 254 nm wavelength (Bintsis et al. 2000). UV-C light is well known for its antimicrobial activity due to its properties affecting the DNA of organisms, causing the formation of DNA pyrimidine-base cross-linkages (cyclobutane pyrimidine dimers and pyrimidine 6–4 pyrimidinone photoproducts), and thus preventing bacterial cell replication and transcription, ultimately resulting in cell death (Sinha and Häder 2002; Ochoa-Velasco et al. 2018).

In the past decade, semi-conductor UV-C light emitting diodes (UV-C LEDs) have emerged as alternative UV-C mercury-free sources, notably for environmental and energy-saving concerns (Song et al. 2016). Moreover, UV-C LEDs can be adjusted in order to emit different unique targeted wavelengths (Chen et al. 2017) and can also present other notable advantages than limiting the risks due to mercury compared to gas-filled lamps: no preliminary warming required, a longer lifespan, a reduced size, a versatile integration into any design, and a low drive voltage requirement (Chen et al. 2017). Therefore, these diodes offer great potential for novel applications in many fields (Kebbi et al. 2020), ranging from water disinfection (Song et al. 2016) to food safety (Kim et al. 2016). For instance, LED lights are promising tools for in situ antimicrobial treatments along water distribution facilities such as plumbing installations and showerheads in order to prevent the development of opportunistic pathogens (Cates and Torkzadeh 2020). In addition, the installation of optical fibres within the plumbing network can provide a treatment solution for the internal surfaces of the pipes, as suggested by (Lanzarini-Lopes et al. 2020), and also for reducing biofouling in membrane filtration systems (Sperle et al. 2020).

Recently, a growing number of studies have now used LEDs as a source of UV for inactivating planktonic (free-floating) bacteria, notably with LEDs emitting at a definite wavelength ranging between 255 and 265 nm, at which DNA absorbs the most (Kim et al. 2016, 2017; Shin et al. 2016; Song et al. 2016). Similar results were obtained by studying the antibacterial effect of LEDs in comparison to a conventional mercury lamp on species such as *Escherichia coli, Listeria monocytogenes*, *Legionella pneumophila*, and *Enterococcus faecalis* among others (Li et al. 2017; Green et al. 2018). On the other hand, studies regarding biofilm eradication by using UV-C LEDs are still scarce, as recently reviewed by Ben Ghorbal et al. (2022). A first study was conducted by applying a 265 nm UV-C LED on *Pseudomonas aeruginosa* biofilms grown for 72 h on catheter-like tubes, and results showed a 6-log reduction in the bacterial population (Bak et al. 2010). Similarly, Argyraki et al. (2017) showed that applying UV-C LED irradiation at 266 nm on a *P. aeruginosa* biofilm growing on an ester cellulose membrane reduced the bacterial biomass by 90% within 24 h. More recently, Gora et al. (2019) also reported about a 1.5 log decrease for *P. aeruginosa* biofilm grown on polycarbonate coupon in a CDC biofilm reactor using a UV-C LED emitting at 268 nm. These results were recently confirmed by a study that compared different wavelengths between 222 and 282 nm on the same type of biofilms with a maturity of between 1 and 5 days. The authors found an efficiency of between 1.5 and 2.5 log reduction, with a maximum effect for young biofilms and after irradiation at 270 nm (Ma et al. 2022).

In general terms, studies on biofilm inactivation are facing issues related to the large diversity of bacterial strains to eradicate, the selection of the most relevant model biofilms (grown under static or dynamic conditions), and the choice of adapted analytical tools, which explains standardization initiatives such as the MiABie, Miminum information About a Biofilm experiment (Lourenço et al. 2014). Despite these obvious facts, neither the microbial load nor the characteristics of the biofilm matrix are currently considered key parameters in terms of standardization. In addition, the need for a standard protocol for UV-C LED studies targeting microorganism inactivation has been requested in order to accurately compare results from different studies (Song et al. 2016). Indeed, exposure characteristics, such as the radiation profile, radiation power, and distance between LED and samples, are found to vary between studies and are not always thoroughly detailed. Notably, several authors have already started to work on a standard method for UV-dose determination using UV-C LEDs (Kheyrandish et al. 2018) and on the definition of experimental conditions and analytical methods (Gora et al. 2019).

Despite the difficulty to compare results provided by different studies, several authors concluded that applying 280 nm wavelength is an optimum choice to achieve high inactivation efficiencies with low energy consumption by acting both on the DNA (although reduced) and proteins (Li et al. 2017, 2019; Rattanakul and Oguma 2018). Indeed, 280 nm is the maximum absorbance peak for proteins, mainly due to the presence of aromatic amino acids, and such a wavelength is known to alter cellular proteins (Santos et al. 2013), including enzymes involved in DNA repair systems (photoreactivation and dark repair) which can hence prevent cell reconstruction after the treatment (Kebbi et al. 2020). Furthermore, Gerchman et al. (2020) have recently shown that a UV-LED with 279 nm peak emissions could offer efficient inactivation of many viruses, including the coronavirus family.

The biofilm matrix of different bacterial strains has been described as rich in proteins (Randrianjatovo et al. 2015), along with extracellular DNA (Whitchurch et al. 2002; Okshevsky and Meyer 2013) and polysaccharides (Wei and Ma 2013). *P. aeruginosa* is the most commonly used microorganism model in biofilm research and was therefore chosen for this study. This ubiquitous motile Gram-negative bacterium is an opportunistic pathogen that can be found in diverse environments and can cause harmful human infections when contaminating food or water supply networks. For instance, its occurrence in drinking water is mainly related to its ability to build up biofilms in plumbing installations (Bédard et al. 2016).

Besides this bacterium, lactic acid bacteria such as *Leuconostoc citreum* can also be used to study biofilms but are seldom found in this research field. Nevertheless, *Leuconostoc citreum* has the specificity to produce high molecular-weight glucan exopolymers from sucrose due to extracellular glucansucrases (Bounaix et al. 2009; Passerini et al. 2015). The involvement of these glucan polymers in biofilm formation has been shown to be essential (Badel et al. 2008; Leathers and Bischoff 2011). Moreover, *L. citreum* can form biofilms, which can have an economic impact on industrial processes (Naessens et al. 2005; Arena et al. 2017).

The objective of the present work was therefore to evaluate the efficacy of a UV-C treatment by using a 280 nm UV-C LED device on two model biofilms produced by *Pseudomonas aeruginosa* and *Leuconostoc citreum* strains. These strains were chosen because they produce quite different matrices in terms of quantity and biochemical composition; moreover, *L. citreum* produces extracellular dextrans when grown in the presence of sucrose, which allows obtaining two different types of matrices for the same strain. This investigation was carried out with particular attention to the inherent properties of each biofilm, such as the initial cell density and the extracellular matrix characteristics. In order to standardize these properties, a preliminary methodology was developed to obtain surface-spread bacteria and biofilms calibrated in terms of cell concentration and matrix composition. Irradiation experiments were performed by varying the duration of exposure, which provided dose–response curves based on log CFU decrease.

## Material and methods

### Bacterial strains and growth conditions

The bacterial strains were chosen considering cell wall structure and principal extracellular matrix components. *Pseudomonas aeruginosa* ATCC 15442 is a Gram-negative strain recommended as a standard model strain for testing antimicrobial disinfectants under national regulatory standards NF EN1040 (AFNOR 2006) and ASTM standards. *Leuconostoc citreum* NRRL B-1299 (= ATCC 11449) is a Gram-positive strain that has the particular characteristic of producing high-molecular weight dextrans (i.e. glucans) in the presence of sucrose (Bounaix et al. 2009; Passerini et al. 2015), and develops biofilms with different thicknesses depending on sucrose availability.

Liquid or solid cultures were prepared from stock cultures for both strains and maintained at -80 °C in glycerol (20% v/v). *P. aeruginosa* was routinely cultivated at 37 °C in LB (Luria Bertani) medium, and *L. citreum* was cultivated at 30 °C in MRS (de Man Rogosa and Sharpe) medium. To induce the production of extracellular polysaccharide, *L. citreum* MRS medium was supplemented with sucrose (40 g/L).

### Sample preparation: surface-spread bacteria and biofilms

**Fig. 1:**
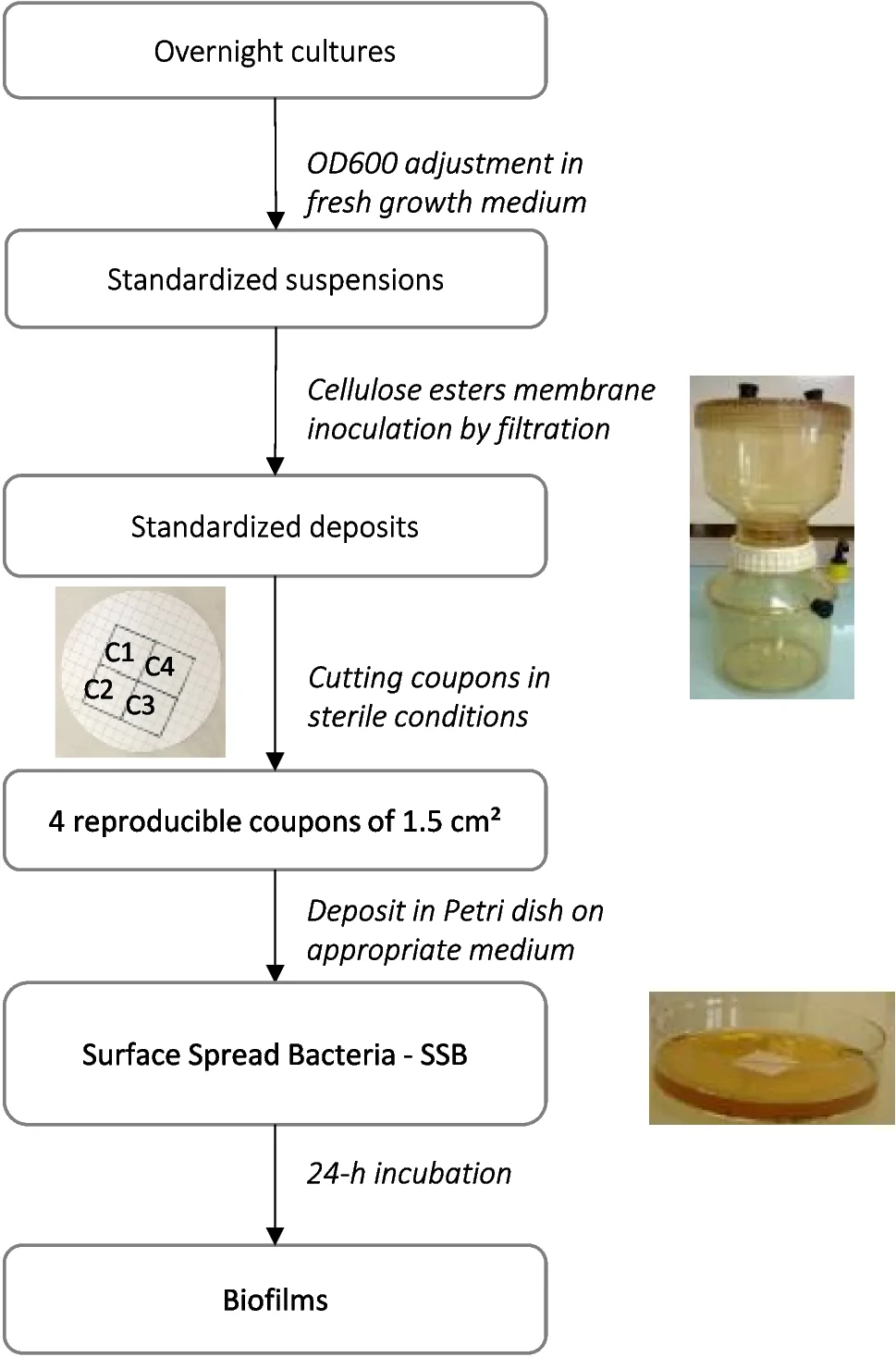
Methodology for sample formation: 24 h-biofilms and surface spread bacteria (SSB) calibrated in cell density

The general outline of the procedure is described in Fig. 1. Biofilms were grown on hydrophilic mixed cellulose ester membranes (with grids) as described by (Labadie et al. 2021). Bacteria were grown overnight in MRS or LB broth according to the strain and further diluted with sterile MRS or LB medium to obtain the appropriate cell density, which was monitored by OD_600nm_ measurement (see details in Results section—Method development), knowing that OD_600nm_ = 1 correspond to 1 × 10^8^ CFU/mL for *L. citreum* and to 4 × 10^7^ CFU/mL for *P. aeruginosa*. Twenty mL of calibrated suspensions were then filtered through Pall GN-6 Metricel® sterile membranes (diameter 47 mm, pore size 0.45 µm) in order to homogeneously deposit the cells over the membrane surface. The membrane was then cut under sterile conditions into four equal squares of 1.5 cm2. Preliminary experiments showed that the cell density measured by the four 1.5 cm2 coupons cut out from the same membrane was not statistically different, whether filter-deposited cells or biofilm bacteria (data not shown). Each individual coupon was then immediately placed in the centre of a Petri dish (55 mm diameter) containing either MRS solid media with or without sucrose (40 g/L) for *L. citreum*, and LB solid media for *P. aeruginosa*. This process gave calibrated Surface-Spread Bacteria (SSB, Fig. S1) samples, which were used directly either for LED treatment or for biofilm growth. For biofilm growth, plates were incubated for 24 h at the appropriate temperature according to the strain. As mentioned above, *L.citreum* can provide two types of biofilms according to the presence or absence of sucrose in the growth medium; the biofilms produced in the presence of sucrose will be called hereafter “glucan-rich” biofilms (GR biofilms).

Biofilm dry weight (DW) measurements were performed with a moisture analyzer Sartorius MA30 and on 24-h biofilms grown on whole 47 mm diameter membranes (*n* = 2). DW measurements were corrected with the mass of the membrane.

### UV-C LED source and sample exposure

**Fig. 2:**
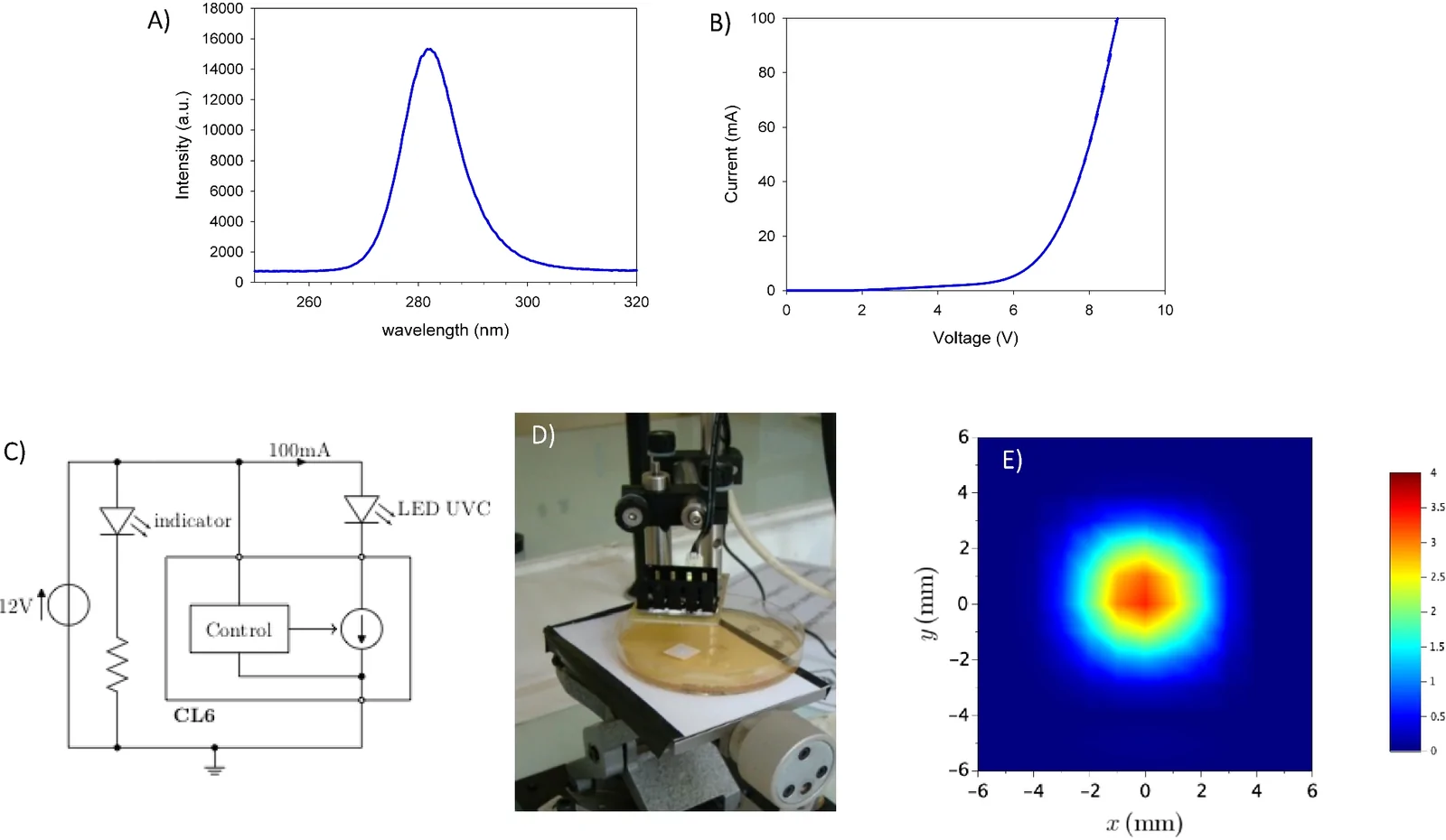
UV-C LED experimental setup. **A** Spectral intensity distribution Electric diagram. **B** Relationship between voltage and current. **C** Electric diagram. **D** Setup unit of the inactivation experiment. **E** Spatial distribution of the irradiance of the UV-LED on the coupon surface

The UV-C treatment was conducted using an Optan UVC-LED (Crystal IS, USA) which emitted at 280 nm (Optan-280 K-BL). A static laboratory-designed UV LED setup unit was implemented with a fixed constant-current linear LED driver (Microchip CL6N5-G) at 100 mA from 12 V power supply, and the LED package was mounted on a heatsink to dissipate the surrounding air heated in the junction of the LED (Fig. 2A). In these conditions, the LED light power output at ambient temperature was 2 mW. The spectral bandwidth was estimated by optical emission spectroscopy (Ocean Optics HR400). The spectral intensity distribution of the LED revealed a maximum emission at 281 nm and a spectral emission full-width at half-maximum (FWHM) of 13 nm (Fig. 2B). The relationship between tension and intensity was measured with a Keithley model 2602A sourcemeter (Fig. 2C).

The distance between the LED and the coupon was kept constant at 2 cm, and the exposure was conducted at ambient temperature. As a precaution (since the light output varied slightly with the LED temperature), a 5 min stabilization phase was applied before carrying out any UV exposure at the centre of the coupon. Of note is that the plate was not moved during the treatment. Three coupons, issued from the same membrane, were exposed for 5, 10, and 20 min, respectively, and the fourth coupon (from the same membrane) was left untreated to estimate the initial bacterial concentration on the membrane (control sample). Three independent membranes were used per treatment. For each treatment, the applied dose (mJ/cm^2^) was evaluated by multiplying the average irradiance (0.57 mW/cm2, see below) by the exposure duration (in seconds).

The average irradiance was experimentally determined using a radiometry method inspired by a protocol for determining the irradiance in a water suspension (Kheyrandish et al. 2018) but simplified because the influence of water must not be taken into account here. The irradiance distribution at the distance of 2 cm from the UV-LED was measured with a spectrometer (Acton Spectra SP 2750, in the Czerny Turner configuration) with 0.75 m focal length equipped with 600 grooves/mm grating. The detection device set at the exit of this spectrophotometer was an ICCD camera (Princeton Instruments PIMAX 512 × 512 pixels). The detection device was calibrated beforehand with an ocean optic DH-3 standard lamp connected to the spectrometer entrance by an optical fiber (UV-silicon LG- 455–020-3). The entrance of the optic fiber was then mounted on a motorized linear stage with three axes (Standa 8MT175) that could move on a planar surface with high resolution (0.01 mm). The incident irradiance was measured 2 cm from the plane of movement of the entrance of the fiber every millimeter on two perpendicularly crossed lines intersecting at the centre of the coupon surface according to a simplified method presented by Bolton et al. (2015).

### Analyses

#### Evaluation of bacterial viability (bacterial enumeration)

Membrane coupons of untreated control and irradiated samples were placed in 1 mL of a sterile saline solution (NaCl 9 g/L) and vigorously mixed by vortexing for 1 min, which disrupted biofilms and released bacterial cells. Serial tenfold dilutions were then promptly made on the obtained bacterial suspensions, 100 µl aliquots were spread plated in duplicate on appropriate culture media described previously, and colony-forming unit (CFU) counts were determined after 24-h-incubation. The efficacy of the initial recovery step was controlled by counting the bacterial cells remaining on the membrane coupon: coupons were once again placed in 1 mL of sterile physiological solution and treated as described above. These experiments showed that more than 95% of cells could be recovered from the coupon by applying the one-step procedure, in agreement with the commercial technical data of the membrane (removal efficiency > 90% for *Escherichia coli)*. This step enabled to easily recover bacterial cells without an additional step such as sonication thereby minimizing any cell damage. Serial dilutions, plate inoculation, and subsequent incubation were performed immediately after UV exposure. In these conditions, light-dependent DNA repair mechanisms (e.g. photoreactivation*)* were minimised, especially as enzymes involved in DNA repair mechanisms could have been altered during the irradiation at 280 nm.

The log reduction value was calculated as log_10_(N_0_/N) to quantify the inactivation efficiency of treatments, where N_0_ is the concentration of microorganisms before treatment (control), and N is the concentration after UV treatment, both evaluated from the same initial entire membrane sample.

#### Scanning electron microscopy

Scanning electron microscopy (SEM) analysis was applied to bacterial biofilms which were produced as described previously (Labadie et al. 2021). Briefly, coupons were removed from agar plates and were desiccated under vacuum for 15 min to 1 h, depending on the samples. Samples were then directly metallized with platinum for image acquisitions. Analysis was performed with a MEB Quanta 250 FEG FEI at the microscopy platform Centre de Microscopie Électronique Appliquée à la Biologie (CMEAB) at Toulouse University.

#### Confocal laser scanning microscopic analysis

In addition to the previous characterization of biofilms by infrared spectroscopy (Labadie et al. 2021), observations by confocal microscopy were carried out on the biofilms before irradiation. Due to autofluorescence of Pall GN-6 Metricel® membrane, confocal laser scanning microscopic (CLSM) analysis was undertaken on *P. aeruginosa* biofilms, which were grown on a black polycarbonate membrane (GTBP Isopore; diameter 13 mm, pore size 0.22 µm). In this case, the membrane was placed on the surface of LB agar medium, and the bacterial inoculation was made by spotting 50 µL of a calibrated suspension (about 3 × 10^6^ CFU/mL), followed by a 10-min drying step in a microbiological safety cabinet. The membrane was then incubated for 24 h at 37 °C to allow biofilm growth. After incubation, the biofilm matrix was co-labelled as previously described in (Randrianjatovo et al. 2015; Randrianjatovo-Gbalou et al. 2017): eDNA and extracellular protein labelling were carried out using 50 µL of 2 μmol/L TOTO-1® for exactly 20 min and epicocconone (Fluoroprofile Protein Quantification kit FP0010) for 30 min, respectively. At the end of labelling, the membrane was mounted in Mowiol® 4–88 (81,381, Sigma-Aldrich), and placed between the slide and a square coverslip (22 × 22 mm) using a Secure-Seal™ spacer 13 mm in diameter and 0.12 mm thick (Invitrogen, and ThermoFisher). The slide was kept overnight at 4 °C in the dark to allow the mounting solution to polymerize and before microscopic observations with a Leica SP2 AOBS confocal laser scanning microscope (Leica Microsystems, France). The CLSM images were obtained using a 63x/0.9NA water immersion lens, and the Leica control software (LCS) was set to take image scans of 512 × 512 pixels (corresponding to 238 by 238 µm) with a speed of 400 Hz. Each channel was scanned between frames with the first excitation of epicocconone by a helium–neon laser at 561 nm, followed by TOTO-1 (argon laser at 488 nm). It should be noted that *L. citreum* biofilms (glucan-rich or not) could not be imaged due to biomass detachment events that occurred when operating the fixing and staining protocol.

#### Statistical analysis

All irradiation experiments were conducted in three or four independent replicates, with independent initial bacterial cultures, and results were expressed as mean ± sd. All statistical analyses were performed using XLSTAT. For cell density groups (biofilms and SSB) and inactivation efficiencies, the Shapiro–Wilk test was used to determine the normal distribution of experimental values (*p* > 0.05), and the homogeneity of variance was assessed using the Levene test (*p* > 0.05). Then, a one-way ANOVA was conducted, and the Newman-Keuls multiple comparison test was used (*p* < 0.05).

GInaFiT (Geeraerd and Van Impe Inactivation Model Fitting Tool), a Microsoft Excel plug-in developed to assess no-log-linear microbial survival curves, was used to model bacterial inactivation (Geeraerd et al. 2005).

In addition, for each strain, a multiple regression analysis was performed to obtain an equation expressing the final CFU counts (CFU_f_) as a function of the type of matrix considered as a binary data for *P. aeruginosa* (absence 0 / presence 1) and an ordered descriptive variable for *L. citreum* (absence 0 / presence without sucrose 1 / presence with sucrose 2*)*, and two quantitative variables: the initial cell load expressed as log_10_(CFU_i_/cm^2^), and the time of LED-UV-C irradiation in min:

$${\mathrm{log}}_{10}({\mathrm{CFU}}_f/\mathrm{cm}^2)\hspace{0.17em}=\hspace{0.17em}\mathrm a\;\mathrm m\mathrm a\mathrm t\mathrm r\mathrm i\mathrm x\hspace{0.17em}+\hspace{0.17em}\mathrm b\;{\mathrm{log}}_{10}({\mathrm{CFU}}_i/\mathrm{cm}^2)\hspace{0.17em}+\hspace{0.17em}\mathrm c\;\mathrm t\mathrm i\mathrm m\mathrm e\hspace{0.17em}+\hspace{0.17em}\mathrm{intercept}$$

Finally, a third test was carried out with all the data, but considering the bacterial strains and the presence of matrix as binary variables:

$${{\mathrm{log}}}_{10} ({{\mathrm{CFU}}}_{f}/{{\mathrm{cm}}}^{2})\hspace{0.17em}=\hspace{0.17em}\mathrm{a \;matrix}\hspace{0.17em}+\hspace{0.17em}\mathrm{b }{{\mathrm{log}}}_{10} ({{\mathrm{CFU}}}_{{\mathrm{i}}}/{{\mathrm{cm}}}^{2})\hspace{0.17em}+\hspace{0.17em}\mathrm{c \;time}\hspace{0.17em}+\mathrm{d \;strain}+\hspace{0.17em}{\mathrm{intercept}}$$

In all cases, a Student t-test was performed to decide whether the model coefficients were significant.

## Results

### Method development: sample preparation with calibrated cell density

In order to achieve reproducible 24-h-old biofilms with a controlled cell density of about 1 × 10^8^ CFU/cm^2^, a first set of preliminary experiments was conducted to determine the exact bacterial concentration of both culture suspensions (*L. citreum and P. aeruginosa*) to be deposited on the mixed ester cellulose membrane (Fig. 1). For this purpose, OD_600nm_ calibrated bacterial suspensions, freshly prepared from an overnight culture of *L. citreum,* were tested at three different cell loads (2 × 10^3^ CFU/cm^2^, 2 × 10^4^ CFU/cm^2^, and 3 × 10^6^ CFU/cm^2^) and incubated for 24 h on MRS agar medium. At the end of the incubation period, the coverage of the membrane was assessed by the naked eye, showing partial coverage (i.e. presence of isolated colonies) for the two lowest cell loads. For the highest cell load (20 mL of the culture suspension calibrated at OD_600nm_ = 0.025), the membrane was completely and homogeneously covered, with a cell coverage reaching about 10^8^ CFU/cm^2^. Owing to the different growth characteristics of the two strains studied here, total membrane coverage for *P. aeruginosa* was reached with an initial cell load of only 4 × 10^5^ CFU/cm^2^ (20 mL of a culture suspension calibrated at OD_600nm_ = 0.01). The methodology applied to produce biofilms on coupons led to accurate, reproducible bacterial coverages (Table 1). Indeed, bacterial concentrations were similar for both *P. aeruginosa* and *L. citreum* biofilms, with concentrations reaching 2.9 × 10^8^ ± 2,4 × 10^8^ CFU/cm^2^ and 1.2 × 10^8^ ± 1.2 × 10^7^ CFU/cm^2^, respectively. Cell densities measured for glucan-rich biofilms of *L. citreum*, produced in the same conditions, showed slightly higher levels (8.2 × 10^**8**^ ± 5.3 × 10^8^ CFU/cm^2^).

**Table 1:** Cell density of 24-h biofilm and surface spread bacteria (SSB) samples and associated statistical groups (*n* = 3 or *n* = 4 depending on the samples). Cell density values (Log CFU/cm^2^) with different letters are significantly different (*p* < 0.05) as measured by the Newman–Keuls test

Strain	Type of sample	Log (CFU/cm^2^)	Group denomination
*L. citreum*	SSB	6.7 ± 0.2 ^C^	Low
	SSB	7.8 ± 0.0 ^DE^	Medium / High
	SSB	8.8 ± 0.0 ^F^	Very high
	Biofilm	8.1 ± 0.0 ^E^	High
	Biofilm	8.8 ± 0.2 ^F^	Very high
	Glucan-rich biofilm	9.0 ± 0.2 ^F^	Very high
*P. aeruginosa*	SSB	5.4 ± 0.3 ^A^	Very very low
	SSB	6.1 ± 0.1 ^B^	Very low
	SSB	7.4 ± 0.1 ^D^	Medium
	SSB	8.2 ± 0.3 ^E^	High
	Biofilm	8.1 ± 0.5^E^	High

Besides these three types of biofilm samples, other samples were prepared (particularly SSB ones) in order to obtain samples with a microbial load similar to that of biofilms. We thus obtained six different modalities for *L. citreum* and five for *P. aeruginosa* (Table 1). This enabled us to statistically assess bacterial inactivation at different cell concentrations, and compare the influence of the type of coverage (SSB and biofilm samples), as well as compare the response of both strains.

### Characterization of biofilms

A naked eye assessment of the biofilm coverage over the coupon clearly indicated different features for each of the three biofilm samples: a rough, slimy aspect for *P. aeruginosa*, a flat surface for *L. citreum*, and a very mucous surface for *L. citreum* grown in the presence of sucrose with a biofilm which even spread beyond the coupon. In addition, dry weight measurements also enabled to specify the different amounts of matrix compounds within each biofilm. *P. aeruginosa* biofilm (2.0 ± 0.3 mg/cm^2^) showed a two-fold DW content compared to *L. citreum* (1.2 ± 0.1 mg/cm^2^). Still, growth with sucrose supplementation led to a fourfold higher DW value (4.7 ± 0.6 mg/cm^2^), thereby attesting to a massive production of glucan polymers from this strain.

**Fig. 3:**
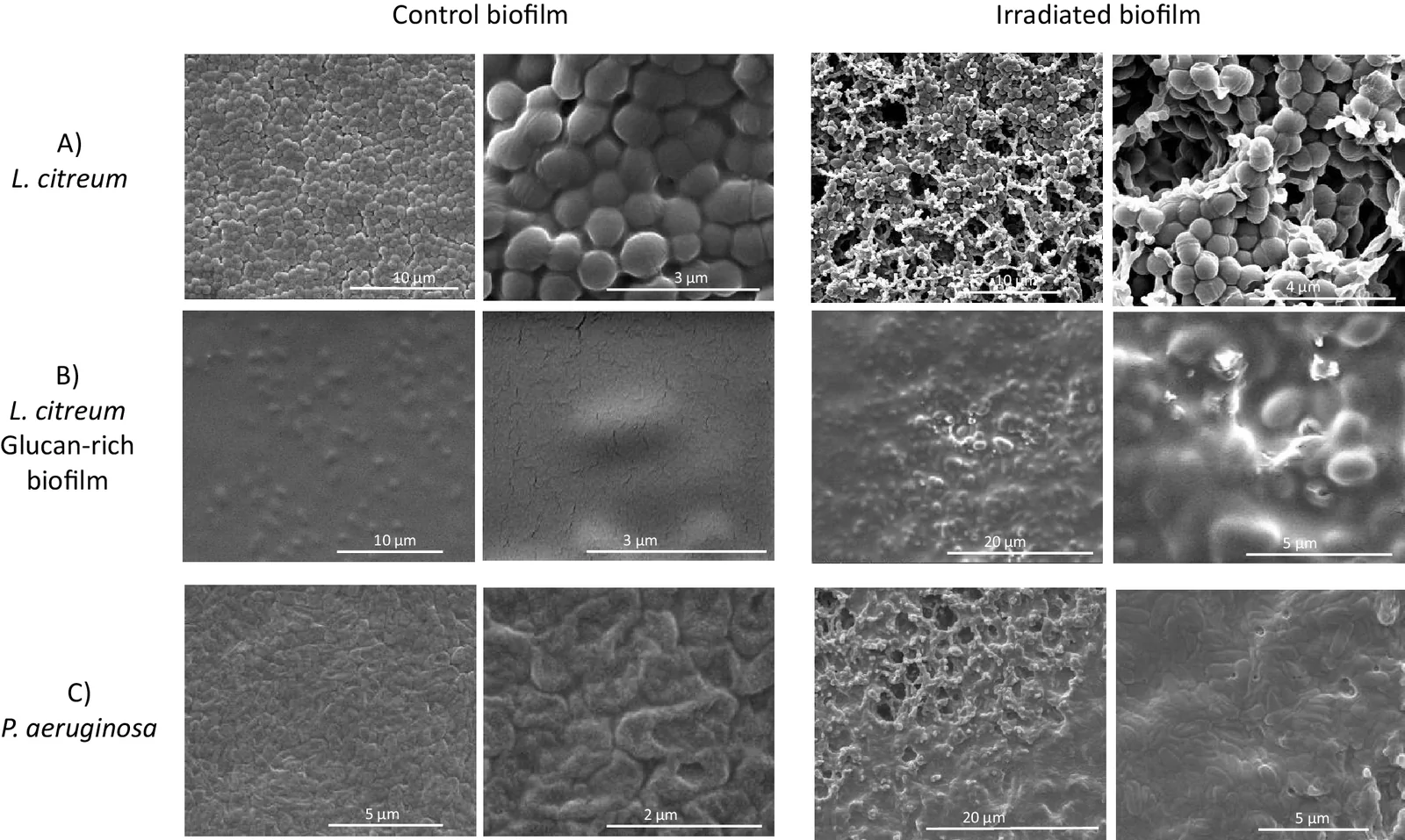
Scanning electron microscopy (SEM) observation of 24-h model biofilms of **A** *L. citreum*, **B** *L. citreum* with induced glucan production, and **C** *P. aeruginosa* at the centre of the coupon. (i) before irradiation at two enlargements: about × 10,000 (first column) and × 30, 000 (second column); (ii) after irradiation (third and fourth columns)

In addition, SEM investigations of each biofilm showed the homogeneous distribution of *L. citreum* and *P. aeruginosa* cells over the membrane coupons, as well as the diversity of each biofilm structure (Fig. 3). Indeed, images showed that all biofilms obtained after 24 h of culture were composed of densely arranged cells covering the whole surface of the membrane. However, biofilms were structured differently: a layer of smooth distinct cells for *L. citreum* biofilm grown on MRS media (Fig. 3A), whilst *L. citreum* cells grown with sucrose-supplemented media were embedded in a much thicker and slimier matrix due to the overproduction of glucans (Fig. 3B), and finally a rough surface for the *P. aeruginosa* biofilm (Fig. 3C).

**Fig. 4:**
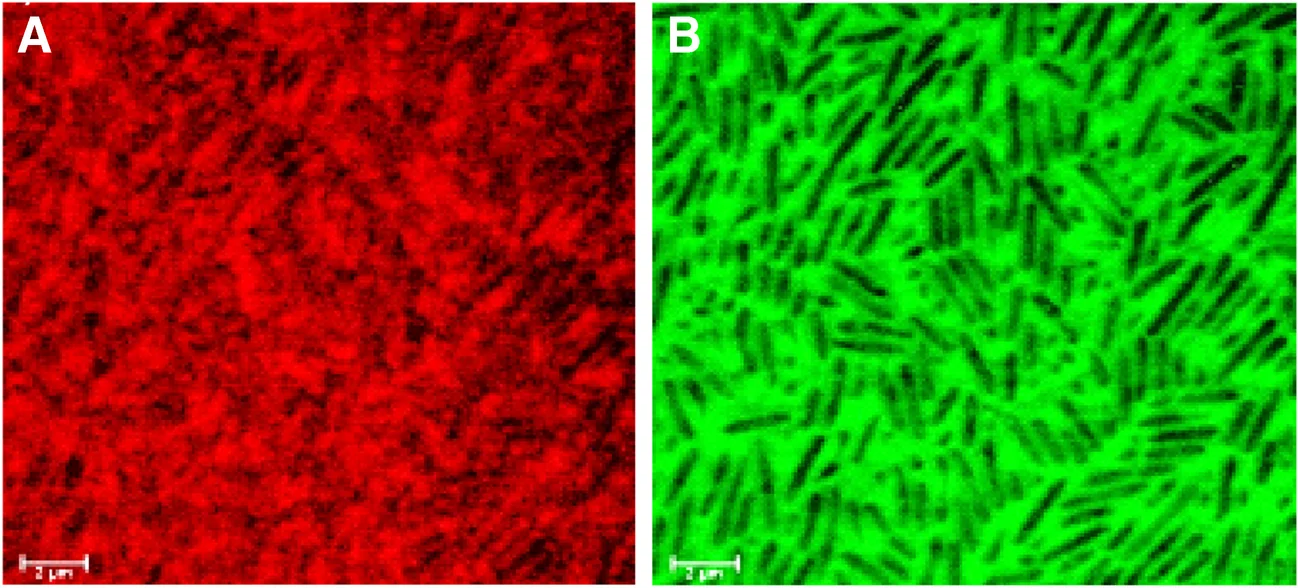
CLSM images of the 24-h biofilm of *P. aeruginosa*. Staining of extracellular **A** proteins using epicocconone, **B** DNA using TOTO-1. (63 × oil immersion objective, Leica SP2 AOBS)

Furthermore, CLSM observations specified the nature of the extracellular matrix of *P. aeruginosa* biofilms before irradiation (Fig. 4). Epicocconone labelling showed that proteins are present and evenly distributed within the biofilm sample (Fig. 4A). Since the labelling was diffuse, it was difficult to clearly differentiate protein components associated with cell walls and those excreted within the matrix. Staining of eDNA with the TOTO-1 marker showed a strong fluorescence response, which occurred exclusively outside bacterial cells (Fig. 4B). Unfortunately, it was not possible to obtain an equivalent imaging of *L. citreum* biofilms, due to biomass detachment from the coupon when the sample went through the fixing and staining procedures.

### Inactivation of surface-spread bacteria and biofilms with UV-C LED

Surface-spread cells and biofilms of *P. aeruginosa* and *L. citreum* were exposed to three irradiation doses (171, 342, and 684 mJ/cm^2^), which correspond to different durations of irradiation (5, 10, and 20 min, respectively), and then compared to an untreated (control) coupon.

#### Inactivation at the highest UV dose

**Fig. 5:**
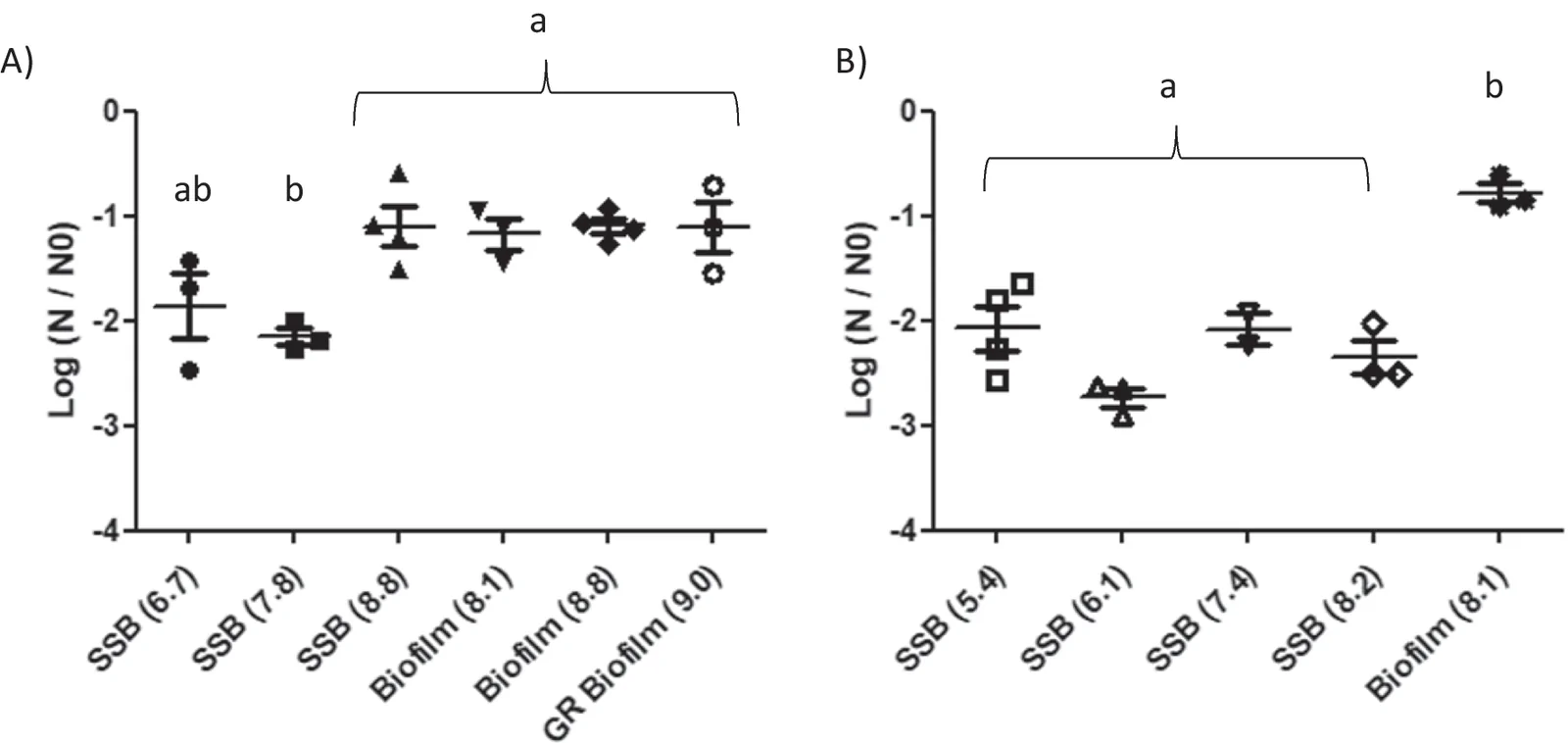
Bacterial inactivation response at 684 mJ/cm^2^ for **A** *Leuconostoc citreum*. **B** *Pseudomonas aeruginosa*. X-axis indicates the sampling type: Surface Spread Bacteria (SSB), Biofilm and Glucan-Rich Biofilm (GR Biofilm) resulting from growth in the presence of sucrose, with the cell load (expressed in log_10_CFU/cm^2^) in parenthesis. Inactivation values with different letters (a, b) are significantly different (*p* < 0.05) as measured by the Newman–Keuls test

**Fig. 6:**
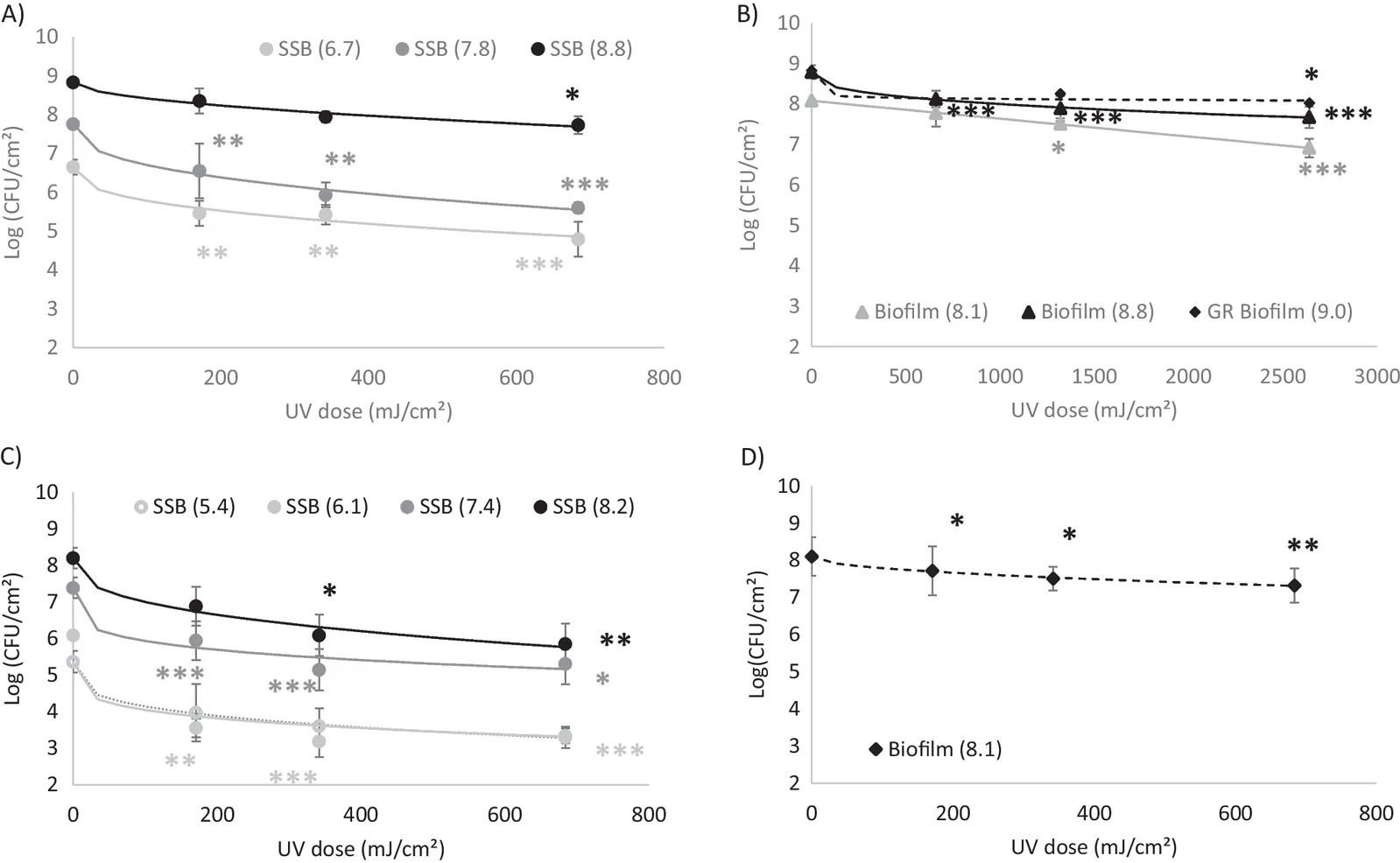
Survival curves of Surface-Spread Bacteria (SSB) and 24-h biofilms after LED UV-C treatment. *L. citreum* SSB **(A**) and biofilms (**B**); *P. aeruginosa* SSB (**C**) and biofilms (**D**) experimental data and Weibull fit. The applied dose was evaluated by multiplying the irradiance by the exposure duration (in seconds). Inactivation was calculated as log_10_ (N/N0); *n* > 3 with error bars indicating standard deviation. Statistical differences from the control (0 mJ/cm^2^) were evaluated by a one-way ANOVA followed by the Newman-Keuls test; *p < 0.05, **p < 0.01, ***p < 0.001

For SSB, a maximum biocide effect of about 2.5 log was observed when applying an irradiation of 684 mJ/cm^2^, and this was similar for both investigated strains (Fig. 5 and Fig. 6).

When considering the effect of the initial cell load on inactivating SSB and over the tested concentration range, results clearly showed that irradiation efficiencies depend on the initial cell density for *L. citreum* (Fig. 5A). Indeed, a 2-log decrease of cell counts was shown for the two lowest cell densities (4.8 × 10^6^ and 5.7 × 10^7^ CFU/cm^2^), while measurements showed only a 1-log population decrease for the highest cell density (Very High group 10^9^ CFU/cm^2^). On the other hand, the effect of the cell density on irradiation efficiency was not observed for surface-spread bacteria of *P. aeruginosa.* Results showed that bacterial inactivation varied between 2.1 and 2.7 log (not significantly different) all over the whole tested concentration range (Fig. 5B). Overall results hence highlight that the initial cell density is an important parameter to take into account when studying biofilm response exposed to UV-C LED treatment.

At a cell density of about 10^8^ CFU/cm^2^ (“high” group), maximal bacterial inactivation was reduced for biofilms compared to SSB (Fig. 5), with log reduction values going from 2.3 ± 0.3 to 0.8 ± 0.2 for *P. aeruginosa* (Fig. 5B) and from 2.2 ± 0.1 to 1.2 ± 0.3 for *L. citreum*. (Fig. 5A). The lower inactivation response obtained for biofilms compared to SSB could attest that the extracellular matrix might have a protective role of inherent cells. This is in line with MEB observations showing that biofilm surfaces changed appearance after irradiation (Fig. 3C), from an initial granular appearance to a smoother and gelatinous state after treatment.

In contrast, the very high cell density of *L. citreum* (10^9^ CFU/cm^2^) showed that there was no statistical evidence of a potential effect of the glucan-rich matrix in response to UV-C irradiation, despite its larger mass (Fig. 5A), with about 1-log reduction value for both SSB and the biofilm forms. Moreover, SEM images showed that no apparent effect was also revealed on the surface morphology of the glucan-rich biofilm (Fig. 3B).

#### Inactivation kinetics—dose effect

When considering the survival curves, it was shown that inactivation response due to irradiation was significant from the smallest applied dose (171 mJ/cm^2^), except for SSBs at maximum cell density; the main inactivation was observed with this dose, and a higher irradiation dose caused only a slight increase in inactivation (Fig. 6).

The UV-dose response data were fitted to a model according to the Weibull frequency distribution (Argyraki et al. 2017). The overall set of data fitted well to the model (*R*2 > 0.93) except for the glucan-rich biofilm (*R*2 = 0.79).

#### Multi-parameters eradication model

For each strain, an in-depth study was carried out based on multiple linear regressions involving the matrix, the initial cell load, and the dose of LED UV-C irradiation. The obtained multiple regressions (Table 2) confirmed the importance of the initial cell density and the dose of treatment on the survival bacterial population*;* these two parameters are indeed very significant (*p* < 0.001) in the model for each strain. In addition, for *P. aeruginosa*, the matrix is also very significant, whereas in the case of *L. citreum*, the biofilm form is only significant at 5%.

**Table 2:** Coefficients of the multiple linear regressions and their significance

	*L. citreum*	*P. aeruginosa*	*Both strains*
Intercept	− 1.728**	− 0.776	− 1.162 **
Dose (mJ/cm2)	− 1.75 10^−3^ ***	− 2.62 10^−3^ ***	2.10 10^−3^ ***
Initial cell load (log(CFU/cm2))	1.168 ***	1.002 ***	1.092 ***
Matrix *P. aeruginosa*		1.102 ***	
Matrix *L. citreum*	0.149 *		
Matrix			0.504 ***
Strain			0.273 *

Significance codes: ***: 0.001; **: 0.01; *: 0.05

**Fig. 7:**
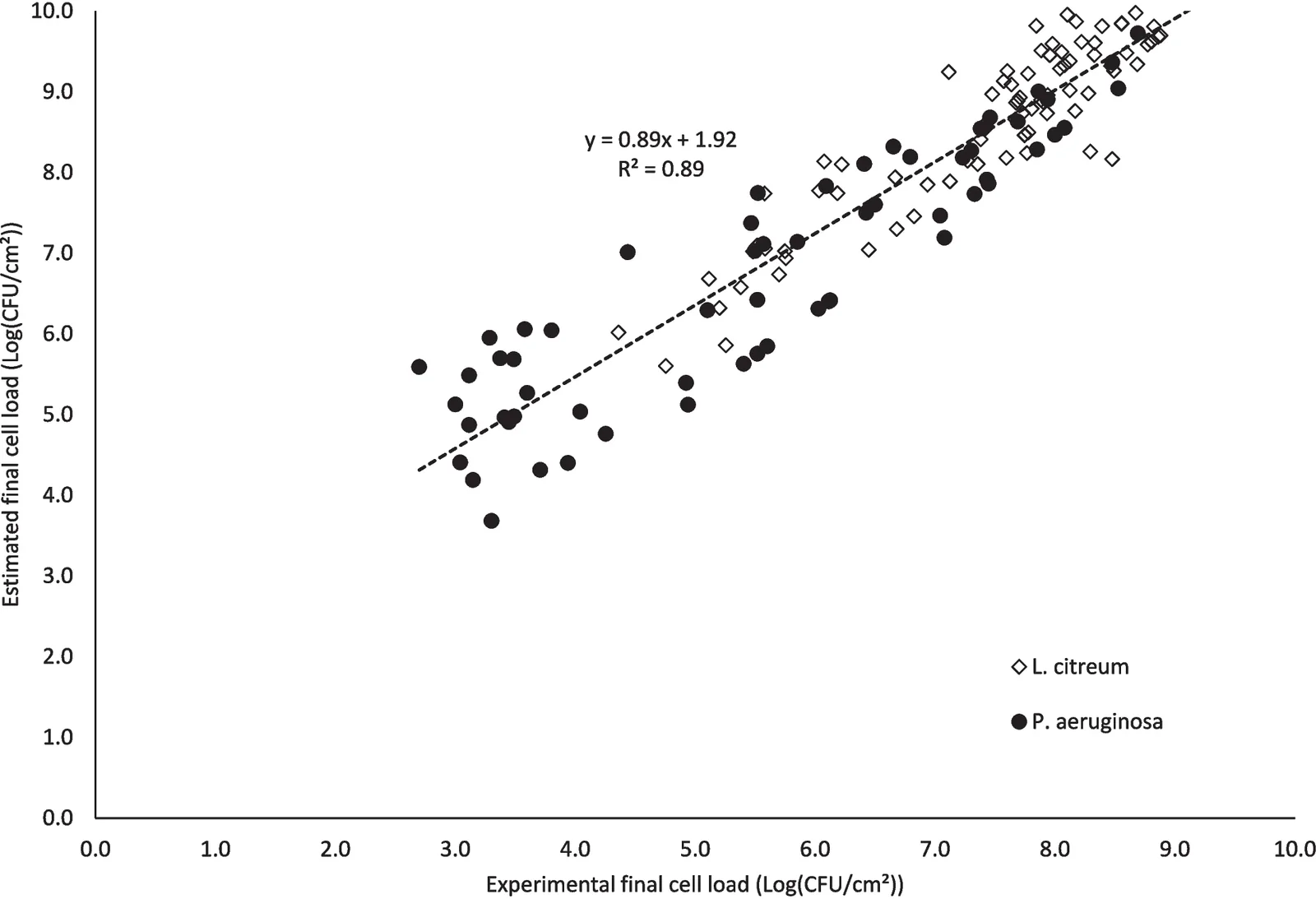
Correlation between estimated cell loads after irradiation (evaluated from the multiple linear regression model) and experimentally obtained cell loads (evaluated by plate counts)

When the matrix was then considered as a binary variable (presence/absence), a final model was established from all the results, enabling to determine the bacterial population after treatment. Subsequent Student *t*-test study showed that the *t-ratios* relative to the five parameters were all significant (Table 2), validating the contribution of each one to the viability reduction, and in particular, the applied dose, the cell density, and the presence of a matrix. Finally, the correlation bringing together the two strains agrees well with the experimental data, with an *R*^2^ value of 0.89 (Fig. 7).

## Discussion

The objective of the present work was to evaluate the efficacy of a UV-C treatment by using a 280 nm UV-C LED device on two model bacterial biofilms and to assess the impact of cell density and the presence of a matrix. A methodology was developed to produce biofilms on coupons with accurate and reproducible bacterial cell loads. This method can enable to study and compare biofilms with similar cell densities and different matrix types, varying quantitatively and qualitatively depending on the strain. Moreover, the bacteria can also be surface-spread in a planktonic state at a defined cell load and further compared to biofilms. At last, this methodology produces biofilms that are easy to transport and to irradiate without detaching the aggregated bacteria from its support. In this study, two bacterial strains, *P. aeruginosa* and *L. citreum*, were chosen considering cell wall structure properties and contrasted matrices in terms of quantity (according to naked eye assessment and dry weight measurements) and biochemical composition. Recent data from ATR-FTIR spectroscopy have provided information on the proteins (based on wave number 1540 cm^−1^, amide II) and the polysaccharides (based on wave number 1070 cm^−1^) composition of these bacterial biofilms (Labadie et al. 2021). *L. citreum* NRRL B-1299 is a Gram-positive strain that has the particular characteristic of producing high-molecular weight dextrans (i.e. glucans) in the presence of sucrose (Bounaix et al. 2009; Passerini et al. 2015). These polymers may represent between 3 and 8% of the biofilm mass (Bounaix et al. 2009). *L. citreum* biofilm (without sucrose) exposed an equilibrated composition between proteins and polysaccharides, with an average amide II/polysaccharides ratio of 1.14 ± 0.04. *L. citreum* biofilms grown with additional sucrose in the media obviously exposed a predominance of extracellular glucans in the matrix (average amide II/polysaccharides ratio of 0.16 ± 0.01) (Labadie et al. 2021). As for the Gram-negative *P. aeruginosa,* biofilms exposed a higher protein content (average amide II/polysaccharides of 1.39 ± 0.03) compared to *L. citreum* (Labadie et al. 2021). CLSM observations in the present study showed proteins uniformly distributed throughout the biofilm sample and also revealed the presence of a large amount of eDNA that was only detected around the bacterial cells. The presence of eDNA in *P. aeruginosa* biofilms is well documented in the literature (Whitchurch et al. 2002), and it has been described to play an important role in the binding, aggregation, and maturation of a biofilm (Okshevsky and Meyer 2013).

Although several authors have already shown that Gram + and Gram- bacteria respond differently to UV-C LED treatments (Shin et al. 2016; Kim et al. 2017), this present study did not show any difference. Indeed, for a similar cell density (“high” groups, about 10^8^ CFU/cm^2^), surface-spread bacteria cell counts decreased similarly for both *P. aeruginosa* and *L. citreum* with a log reduction value of 2.3 ± 0.3 and 2.2 ± 0.1 respectively (Fig. 5). The same trend was also observed for lower cell concentrations, although the same cell loads between both strains could not be achieved. Indeed, besides the Gram trait (mostly related to cell-wall thickness), sensitivity to UV-C radiation and the ability to repair photodamages are both variable depending on bacterial species or even strains (Schenk et al. 2011; Santos et al. 2013; Gwynne and Gallagher 2018). The present results obtained at the highest cell load of *L. citreum* (about 10^9^ CFU/cm^2^) clearly showed that irradiation efficiencies depend on the initial cell density, which has never been reported before, to our knowledge.

At a cell density of about 1 × 10^8^ CFU/cm^2^ (“high” group), maximal bacterial inactivation was reduced for biofilms compared to SSB with log reduction values of 0.8 ± 0.2 and 1.2 ± 0.3 for *P. aeruginosa* and *L. citreum,* respectively). Similarly, cell concentration decreased by 1 log when studying *P. aeruginosa* biofilms grown after 24 h on ester cellulose membranes after a UV-dose irradiation of 2000 mJ/cm^2^ at 266 nm (Argyraki et al. 2017). On the other hand, due to different experimental conditions applied in other studies, results presently obtained diverged from previous works: (i) slightly higher 3-log inactivation was reported when applying a 280 nm UV-C LED treatment on *P. aeruginosa* suspensions, however with a UV dose of only 6 mJ/cm^2^ (Rattanakul and Oguma 2018); (ii) Gora et al. (2019) reported a log reduction value of 1.4 ± 0.3 with *P. aeruginosa* biofilms grown at 10^7^ CFU/cm^2^ after a 8 mJ/cm^2^ irradiation treatment. Here, it clearly appears that comparing results between different studies is a difficult task since the operating conditions vary widely.

The lower inactivation response obtained for biofilms compared to SSB could attest that the extracellular matrix might have a protective role of inherent cells. CLSM observations showed that the extracellular matrix of *P. aeruginosa* ATCC 15442 biofilm was particularly rich in proteins and eDNA. It is well known that DNA is the main cellular target of UV-C, and, as an example, (Santos et al. 2013) already showed that DNA strand breakage was a good predictor of planktonic cell inactivation by UV. Moreover, proteins can also be a target for UV-C radiations that induce some conformational or compositional changes (Santos et al. 2013). As a consequence, eDNA and proteins might have interacted with the UV-C irradiation and absorbed a considerable part of the energy delivered by the LED, thereby protecting bacterial cells from inactivation. SEM observations showing that biofilm surfaces changed appearance after irradiation can suggest that the biochemical composition of the matrix (presumably the protein and eDNA fractions) was modified under 280 nm irradiation treatments. On the contrary, the presence of extracellular glucans produced by *L. citreum* did not modify the inactivation response and hence did not provide a protective effect. The absence of a protective effect when glucans are predominant in a biofilm matrix can be related to the fact that the produced glucans absorb little at 280 nm; indeed, the polymers were extracted from glucan-rich biofilms using the protocol previously described by Bounaix et al. (2009), and spectrophotometry showed non-significant absorbance at the UV-C wavelength.

Moreover, the inactivation kinetics showed a dose effect, which is in agreement with other reports highlighting a two-phase response from adhered bacteria or biofilms when exposed to UV-C LED. This type of response leads to a maximum inactivation level, which cannot be exceeded even if the duration of the treatment is extended (Argyraki et al. 2017; Kim et al. 2017; Gabriel et al. 2018). The present data fitted well to the Weibull frequency distribution, and the outcome of this model assumes that a part of the population might be resistant to the applied UV treatment (Mafart et al. 2002). In the literature, different potential causes of a tailing effect have been mentioned, among which the presence of resistant sub-populations, cell aggregation, non-homogeneity of UV treatment, or even substratum properties (Schenk et al. 2011). In the present study, it was shown that the spatial distribution of the irradiance is not uniform, varying from 3.4 mW/cm^2^ at the centre of the coupon to 0.17 mW/cm^2^ at the edge of the coupon (and thus an average irradiance on the surface of the 1.5 cm^2^ coupon found to be equal to 0.57 mW/cm^2^). This spatial irregularity of the irradiance, due to the small angle of view of the used UV-C LED (15°), could have limited the inactivation response on the edges of the biofilm coupon compared to the central part. Different solutions can be considered and/or combined to improve the treatment efficiency: using an LED with a wider radiation pattern, a device with multiple LEDs (Kim et al. 2017; Akgün and Ünlütürk 2017; Rattanakul and Oguma 2018), pulsed mode application (Nyangaresi et al. 2019), and combination of UV LEDs with different peak emissions (Chevremont et al. 2012; Li et al. 2017; Green et al. 2018).

Lastly, multiple linear regressions were carried out involving the matrix, the initial cell load, and the dose of LED UV-C irradiation. The obtained correlations fit well with the experimental data and clearly show that the biofilm matrix affects the amount of UV irradiation required to inactivate the bacterial cells. This would be due to the shielding effect and/or absorption of a substantial part of the energy by some EPS, thereby preventing the penetration of photons throughout the biofilm’s deepest layers. For example, Ma et al. (2022) have shown spectrophotometrically that a *P. aeruginosa* biofilm absorbs radiation between 230 and 300 nm. For achieving the same 0.8 log inactivation, with the same 10^8^ CFU/cm^2^ initial cell density, a 25 times higher UV-dose was required for *P. aeruginosa* biofilm compared to SSB; for *L. citreum*, a six times higher dose was needed. Such results hence emphasize that it is possible to adjust the UV-C LED dose, which needs to be applied depending on the cell density and the composition of the matrix in the case of biofilms, thereby leading to interesting predictive and applicative solutions. To our knowledge, this is the very first study bringing evidence that both the initial cell load and exopolymeric substances are factors influencing UV-C LED antibiofilm treatment efficacy.

In the present study, a 280 nm UV-C LED irradiation was applied on surface-spread cells and biofilms of *L. citreum* and *P. aeruginosa* under standardized conditions in order to control and compare the influence of the initial cell surface density and the presence of extracellular matrixes. Results clearly showed that this innovative technology could indeed inactivate bacterial strains, with a reduced efficacy on biofilms in comparison to surface-spread bacteria.

This study also revealed a dose-dependent response when applying the irradiation treatment since the inactivation level was highest for the highest UV-C dose. When UV-C LED treatments were applied on biofilms grown with the same initial cell load, its efficacy was reduced whatever the strain. *P. aeruginosa* cell inactivation was particularly reduced due to the presence of extracellular DNA and proteins, hence suggesting a protective effect. Moreover, cell inactivation could be modelled taking into account the matrix, the initial cell load, and the dose of LED UV-C irradiation.

Further studies are now required to gain further knowledge on 280 nm UV-C LED efficacy on other types of bacterial biofilms, notably in the first stages of biofilm formation in the perspective of preventive applications. Again, in terms of future studies, there would be a need to optimize the designed equipment, and the terms and conditions of use in order to determine and reach the required energy–dose to be distributed per unit area in order to achieve higher levels of biofilm removal. Such achievements would encourage the use of UV-C LED technology in biofilm eradication throughout a large range of applications such as for water disinfection systems as well as biofouling removal.

## Supplementary Information

Below is the link to the electronic supplementary material.

Supplementary file1 (PDF 125 KB)

## Data Availability

The data that support the findings of this study are not openly available due to reasons of sensitivity and are available from the corresponding author upon reasonable request.

## Declarations

This article does not contain any studies with human participants or animals performed by any of the authors.
