# Supplementary material for: Cell density and extracellular matrix composition mitigate bacterial biofilm sensitivity to UV-C LED irradiation
Source: Appl Microbiol Biotechnol. 2024 Apr 5;108:286. doi: 10.1007/s00253-024-13123-4 (PMC10997551; doi:10.1007/s00253-024-13123-4)
Supplement: Supplementary file 1 [file 253_2024_13123_MOESM1_ESM.pdf]

AMAB-D-23-01005**R1**.

entitled ‘Cell density and extracellular matrix composition mitigate bacterial biofilm sensitivity to UV-C LED irradiation.”

by Maritxu Labadie, Frédéric Marchal, Nofel Merbahi, Elisabeth Girbal-Neuhauser, Catherine Fontagné-Faucher and Claire-Emmanuelle Marcato-Romain

Supplementary Information. Surface-Spread Bacteria (SSB) study

A) *L. citreum* B) *P. aeruginosa*

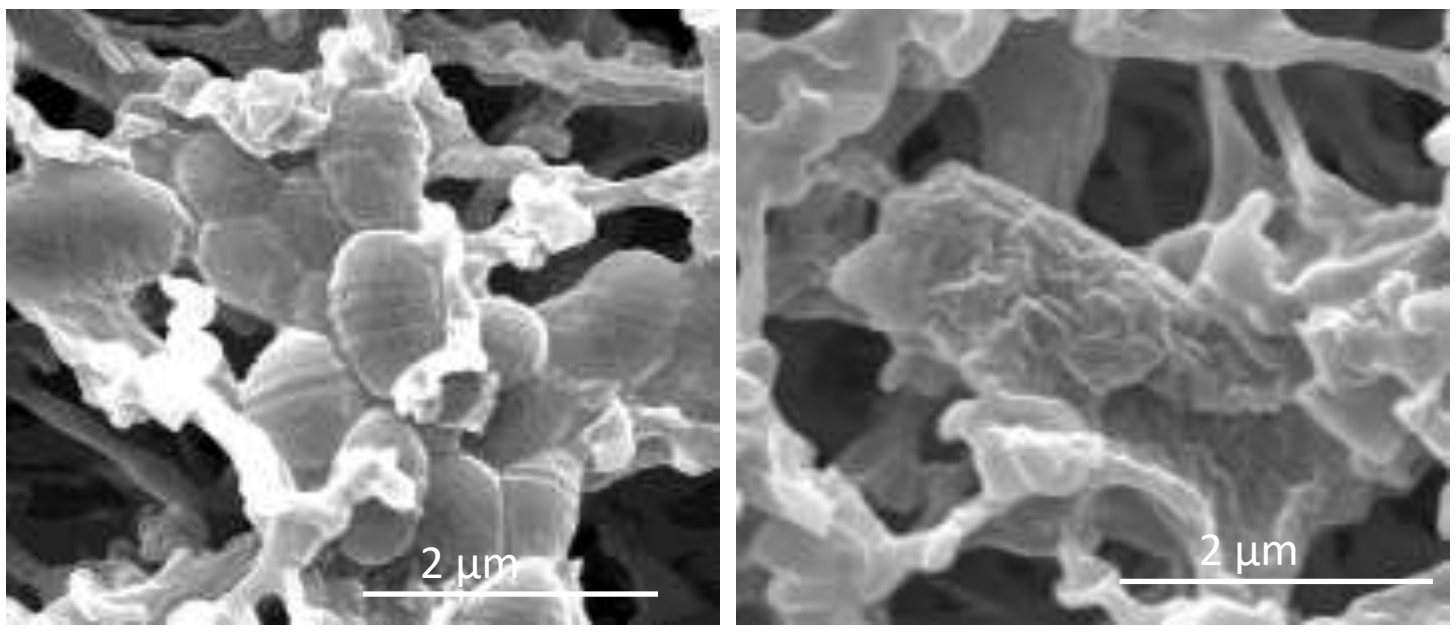

Figure S1. Scanning electron microscopy (SEM) observation of Surface Spread Bacteria (SSB) samples of A) *L. citreum,* and B) *P. aeruginosa* at the centre of the coupon. Magnification about x30,000. The main purpose of these SEM observations was to assess the size of the bacterial cell and to visualise their surface, under the experimental conditions used. Images showed that individual cells are embedded in the membrane network, with no apparent matrix surrounding the cells. Results previously obtained by ATR-FTIR spectroscopy provided qualitative information on the nature of the extracellular compounds for these planktonic forms of the two bacteria (Labadie et. al, 2021).
